# Reevaluating Projections: Total Knee Arthroplasty Volume Before and After COVID‐19 in a Single Orthopedic Practice

**DOI:** 10.1155/aort/5527598

**Published:** 2026-10-06

**Authors:** Mitchel Hawley, Lachlan F. Anderson, Erica R. Olfson, David N. Shau

**Affiliations:** ^1^ Anne Burnett Marion School of Medicine Texas Christian University Fort Worth Texas USA tcu.edu; ^2^ Texas Hip and Knee Foundation Fort Worth Texas USA; ^3^ Texas Hip and Knee Center Fort Worth Texas USA

**Keywords:** arthroplasty, COVID-19, healthcare utilization, knee, replacement, retrospective studies, revision knee arthroplasty

## Abstract

**Introduction:**

The increasing demand for primary (pTKA) and revision total knee arthroplasty (rTKA) has been well documented, with previous literature projecting growth through the year 2030. However, the COVID‐19 pandemic introduced a significant disruption to healthcare delivery, and its long‐term impact on arthroplasty volume trends remains unclear. This study examines the trajectory of pTKA and rTKA volumes in a high‐volume private orthopedic practice before and after the pandemic to assess potential lasting effects.

**Methods:**

This retrospective observational study included 11,589 pTKA and 857 rTKA procedures performed between January 1, 2018, and December 31, 2023. Quarterly volumes were analyzed using segmented interrupted time‐series regression, with Q2 2020 (April–June) defined as the interruption. Models estimated the preinterruption trend, immediate level change, change in slope, and postinterruption trend using Newey–West standard errors. Sensitivity analyses excluded Q2 2020, modeled it as a temporary shutdown, and adjusted for calendar‐quarter seasonality.

**Results:**

The preinterruption pTKA trend was +1.30 procedures per quarter (95% CI, −6.61–9.21; *p* = 0.735). At Q2 2020, pTKA volume decreased immediately by 90.33 procedures (95% CI, −156.21–−24.45; *p* = 0.010). The postinterruption slope was +11.67 procedures per quarter (95% CI, 4.93–18.40; *p* = 0.002); however, the change from the preinterruption slope was not statistically significant (+10.37 procedures per quarter; 95% CI, −2.08–22.82; *p* = 0.098). The positive postrecovery pTKA slope persisted after excluding Q2 2020. No significant immediate level or slope changes were identified for rTKA, including a postinterruption slope of +0.63 procedures per quarter (95% CI, −0.68–1.93; *p* = 0.328).

**Conclusion:**

Within this single private orthopedic practice, the pandemic interruption was associated with an immediate reduction in pTKA volume followed by a positive postinterruption trend. However, the formal change from the pre‐interruption slope was not statistically significant, and no significant trend changes were identified for rTKA. These findings represent temporal associations and may also reflect deferred care, outpatient migration, expanded ambulatory capacity, and other contemporaneous practice or policy changes.

## 1. Introduction

Primary total knee arthroplasty (pTKA) is among one of the most frequently performed elective surgical procedures in the U.S., with over 1.2 million pTKAs reported from 2012 to 2020 [1–4]. The aging population, coupled with an increase in conditions necessitating pTKA, is expected to boost the annual incidence of the procedure. Modeling projections using various statistical analysis methods such as Poisson regression by Sloan et al. in 2018 predicted an 85% increase in the number of annual pTKA by 2030, while a 2019 estimate by Singh et al. projected a 401% increase in procedures by 2040 [5–7]. Earlier projections by Kurtz et al. similarly demonstrated substantial growth in primary and revision knee arthroplasty volumes, using national, administrative, and publicly published data, establishing the current foundation of arthroplasty forecasting approaches [8]. More recent modules have also expanded upon this foundation by incorporating age and sex stratified trends to better illustrate current arthroplasty demands [9]. Of note, more advanced statistical models such as the HIP‐IMPACT have not been employed in this setting.

Despite the positive impact these surgeries have on patients, there are complication risks associated with pTKA, most commonly prosthetic instability, prosthetic joint infection (PJI), and aseptic loosening [5]. Therefore, the rise in pTKA is inevitably accompanied by an increase in the number of revision total knee arthroplasty (rTKA) procedures performed. The number of rTKA procedures was projected to increase at a similar rate to pTKA, with procedure incidence estimated to grow 78%–182% annually [5].

A poorly investigated factor influencing the volume of pTKA and rTKA surgeries in recent years is the COVID‐19 pandemic. Early institutional reports described rapid restructuring of orthopedic services during SARS‐CoV‐2, including implementation of new perioperative safety protocols and postponement of indicated operations when patients’ COVID‐19 status worsened, underscoring how infection‐control demands directly constrained operative throughput [10]. It has, therefore, been reported across all analyzed elective surgical cases that the pandemic caused the largest percentage decrease in orthopedic arthroplasty procedure volume [11, 12]. Specifically, this led to a significant reduction in the number of elective TKAs performed, doubling delay time for pTKA and diminishing the quality of life in these patients [11, 12]. The projected backlog of cases due to the pandemic was estimated to take between 9 and 35 months to recover, with the increased wait times leading to an increase in anxiety due to the uncertainty of when they would receive care [12]. Financially, there was a 17.6% decrease in total surgeon’s charges for TKA procedures and total hip arthroplasty procedures, leading to an estimated loss of $24.4 million in the pandemic year [13]. Furthermore, monthly hospital reimbursement was found to have a statistically significant (*p* < 0.005) decline from an average of $6,136,772 (SD = 2,471,386) in the prepandemic period to $4,855,621 (SD = 2,243,857) in the pandemic year [13].

Therefore, the purpose of this study is twofold: (1) to characterize established volume trends for pTKA and rTKA in a large, high‐volume orthopedic arthroplasty practice prior to the COVID‐19 pandemic and (2) to analyze the impact of the pandemic on these trends through three years of follow‐up. By evaluating both pre and post pandemic data, this study provides insight into how the COVID‐19 pandemic has shaped arthroplasty practice patterns and informs strategies for managing rising demand in the future.

## 2. Methods

### 2.1. Study Design and Setting

This retrospective observational study evaluated pTKA and rTKA volumes within a high‐volume private orthopedic practice. The practice consisted of seven orthopedic surgeons in 2018 and 2019 and eight surgeons from 2020 through 2023, all fellowship‐trained, performing procedures at both hospital and ambulatory surgery‐center locations.

All pTKA and rTKA procedures performed by the practice between January 1, 2018, and December 31, 2023, were eligible for inclusion. Procedures other than primary or revision knee arthroplasty were excluded. The final cohort included 12,446 procedures, comprising 11,589 pTKAs and 857 rTKAs. Each qualifying operative encounter was counted as a separate procedure. Procedures done on bilateral knees simultaneously were counted as two separate procedures.

This study was reviewed by the University of Texas Southwestern Medical Center Institutional Review Board and was determined to be exempt. Because the study involved retrospective, minimal‐risk analysis of existing patient records, the requirement for individual informed consent was waived by the Institutional Review Board. A STROBE checklist was completed and is provided in the supporting material (Supporting Table 1).

### 2.2. Data Collection and Revision Classification

Procedural data were obtained by querying the practice’s internal Epic electronic health‐record database using prespecified International Classification of Diseases, 10th Revision (ICD‐10) codes for pTKA and rTKA. The codes used to identify procedures and classify revision indications are provided in the supplemental materials. No external database linkage was performed.

Indications for rTKA were classified as prosthetic loosening, PJI, prosthetic instability, prosthetic breakage, periprosthetic fracture, osteolysis, bearing‐surface wear, other mechanical complications, or other nonclassified indications. The “other mechanical” category included implant‐related mechanical complications that could not be classified into one of the prespecified mechanical categories. The “other” category included nonspecific or nonmechanical prosthesis‐related diagnoses that did not align with the predefined groups.

Procedures with a confirmed revision status but an unavailable specific indication were retained in the overall rTKA volume analysis and excluded only from indication‐specific analyses. No missing values were imputed.

### 2.3. Variables and Study Timeline

The primary outcomes were quarterly pTKA and rTKA procedural volumes. Secondary variables included annual procedural volume, year‐over‐year percentage change, surgical facility type (hospital versus ambulatory surgery center), admission status (inpatient versus outpatient), and rTKA indication.

The prepandemic period was defined as January 1, 2018, through March 31, 2020, and the postpandemic period was defined as April 1, 2020, through December 31, 2023. Procedural volumes were aggregated by calendar quarter to reduce short‐term month‐to‐month variability. Calendar Q2 was defined as April through June.

Because all eligible procedures performed during the prespecified six‐year period were included, no a priori sample‐size calculation was performed.

### 2.4. Statistical Analysis

Annual and quarterly procedural volumes were summarized using frequencies, and year‐over‐year percentage changes were calculated. Quarterly pTKA and rTKA volumes were analyzed separately using segmented interrupted time‐series regression, with Q2 2020 defined as the interruption. Models estimated the preinterruption trend, immediate level change, change in slope, and postinterruption trend using Newey–West standard errors with a one‐quarter lag. Autocorrelation was assessed using residual autocorrelation plots and the Durbin–Watson test. Sensitivity analyses excluded Q2 2020, modeled it as a temporary shutdown, and adjusted for calendar‐quarter seasonality. The preinterruption trend was also extrapolated to compare projected and observed postinterruption volumes. Estimates are reported with 95% confidence intervals and two‐sided *p* values, with *p* < 0.05 considered significant. Analyses were conducted using R Version 4.4.

## 3. Results

### 3.1. Demographics and Volume

The total number of cases reviewed was 12,446 cases with a total of 11,589 pTKAs and 857 rTKAs. The distribution of pTKAs and rTKAs across the six‐year period is presented in Figure 1. The volume data are further stratified by calendar quarter in Figure 2.

**FIGURE 1:**
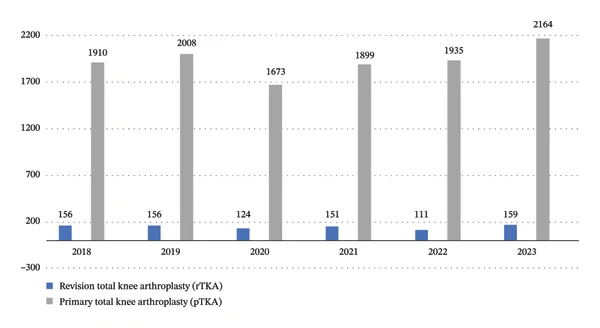
Volume of primary total knee arthroplasty and revision total knee arthroplasty between 2018 and 2023.

**FIGURE 2:**
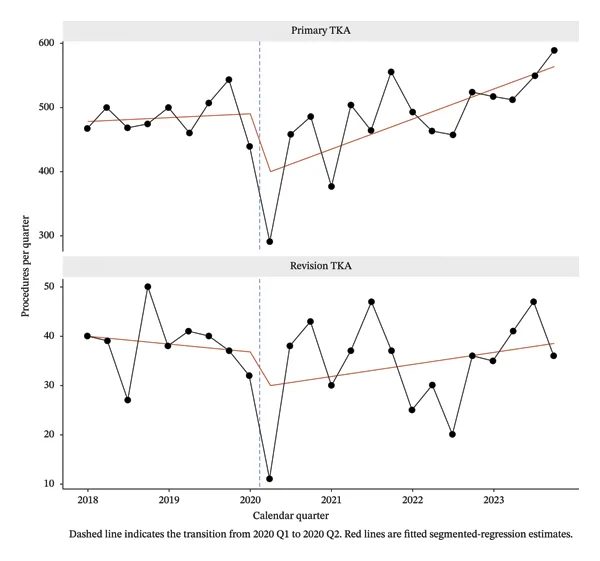
Segmented interrupted time‐series analysis of quarterly primary and revision total knee arthroplasty volumes before and after the COVID‐19 interruption.

### 3.2. Trends in the Number of pTKA and rTKA

As demonstrated in Figure 1, the number of pTKAs increased by 13.3% over the six‐year study period, from 1910 in 2018 to 2164 in 2023, showing an average annual increase of 2.66%. While the overall trend showed growth, there was a notable 16.7% decrease in pTKAs between 2019 and 2020. Following this decline, a slight plateau was observed between 2021 and 2022, with only a 1.9% increase, succeeded by a more substantial 11.8% rise in 2023. Notably, when looking at the post‐2020 data, there was a 29.35% increase in procedural volume, with an average yearly percent increase of 9.08%.

In contrast, the number of rTKAs demonstrated more variability over the six‐year period, with a modest overall increase of 1.9%, from 156 to 159 procedures. Mirroring the trend seen in pTKAs, rTKAs experienced a 20.5% reduction from 2019 to 2020, followed by a 21.8% rebound in 2021. However, volumes dropped again in 2022 by 26.5% before increasing by 43.2% in 2023 (Table 1).

**TABLE 1:** Primary and revision total knee arthroplasty volume from 2018 to 2023 with yearly percent change.

Year	Primary TKA (*n*)	Annual change (%)	Revision TKA (*n*)	Annual change (%)
2018	1910	—	156	—
2019	2008	5.13	156	0.00
2020	1673	−16.68	124	−20.51
2021	1899	13.51	151	21.77
2022	1935	1.90	111	−26.49
2023	2164	11.83	159	43.24
Total	**11,589**	—	**857**	—

*Note:* Bolded values are the cumulative totals of primary and revision TKAs throughout the study period.

Segmented interrupted time‐series analysis demonstrated that the preinterruption pTKA trend was not statistically significant (+1.30 procedures per quarter; 95% CI, −6.61–9.21; *p* = 0.735). At Q2 2020, pTKA volume decreased immediately by an estimated 90.33 procedures (95% CI, −156.21–−24.45; *p* = 0.010). The postinterruption slope was +11.67 procedures per quarter (95% CI, 4.93–18.40; *p* = 0.002); however, the change from the preinterruption slope was not statistically significant (+10.37 procedures per quarter; 95% CI, −2.08–22.82; *p* = 0.098) (Figure 2). When Q2 2020 was excluded, the postrecovery pTKA slope remained positive (+8.06 procedures per quarter; 95% CI, 3.60–12.52; *p* = 0.001) although the change from the prerecovery slope remained nonsignificant (*p* = 0.123). In the seasonality‐adjusted model, the change in the pTKA slope was +11.50 procedures per quarter (95% CI, 0.99–22.01; *p* = 0.034). For rTKA, neither the preinterruption slope (−0.35 procedures per quarter; 95% CI, −1.48–0.78; *p* = 0.525), immediate Q2 2020 level change (−6.65 procedures; 95% CI, −18.53–5.23; *p* = 0.257), change in slope (+0.98 procedures per quarter; 95% CI, −0.96–2.91; *p* = 0.307), nor postinterruption slope (+0.63 procedures per quarter; 95% CI, −0.68–1.93; *p* = 0.328) was statistically significant. Sensitivity analysis modeling Q2 2020 as a temporary shutdown identified an acute reduction of 199.72 pTKAs and 25.47 rTKAs during that quarter (both *p* < 0.001). No significant positive residual autocorrelation was detected for either model.

### 3.3. Trends in Quarterly pTKA and rTKA Volume With Respect to COVID‐19

These data demonstrated a decrease in surgical volume from 2019 to 2020, coinciding with the onset of the COVID‐19 pandemic and associated restrictions. In the first quarter of 2020, defined as “Pre‐COVID,” pTKA volume was 14.3% lower than in Q4 2019. The decrease began in March, affecting Q1 volume, and peaked in April, during which no pTKAs or rTKAs were performed. Cases resumed in May, and by June, total pTKA and rTKA volumes exceeded those observed immediately before the onset of the COVID‐19 pandemic, as shown in Figure 2.

### 3.4. Trends in the Locations of pTKA and rTKA

From 2018 to 2023, there was a large increase in the number of pTKAs being performed at an outpatient, ambulatory surgery center. This specific orthopedic surgery group began utilizing a local surgery center that opened in 2020 resulting in a total of 24 pTKAs performed at the surgery center in 2020 and 225 pTKAs performed at the surgery center in 2023 (Figure 3). As seen in Figure 3, the volume of pTKAs at the hospital facilities stayed relatively constant over the 6‐year period; however, there was a general increase in pTKAs performed at the surgery center across the 4‐year period after the COVID‐19 pandemic.

**FIGURE 3:**
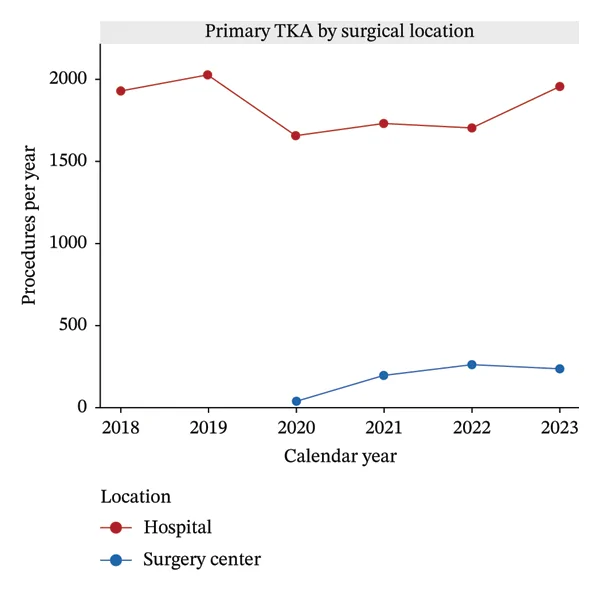
Yearly volume of primary total knee arthroplasty performed in hospital vs. outpatient surgery center from 2018 to 2023.

### 3.5. Trends in Inpatient and Outpatient pTKA Volume

Throughout the study period of January 1st, 2018, through December 31st, 2023, there was a substantial increase in the amount of pTKAs and rTKA performed in the outpatient setting, in comparison to inpatient (Figure 4). In 2018, immediately following January 1st 2018, legislature change which removed TKAs from the Medicare Inpatient Only List (IPOL). In 2018, there were 717 pTKAs performed inpatient and by the end of the study period in 2023, there were only 9 pTKAs performed inpatient through the course of that year. In comparison, 1193 pTKAs were performed outpatient in 2018, and a total of 2155 pTKAs were performed outpatient in 2023. As seen in Figure 4, inpatient TKAs have plateaued with minimal volume annually since 2020; however, outpatient TKAs have grown in volume nearly every year except 2020. In this scenario, it is important to consider the large‐scale impact the COVID‐19 pandemic had on all elective procedures in 2020.

**FIGURE 4:**
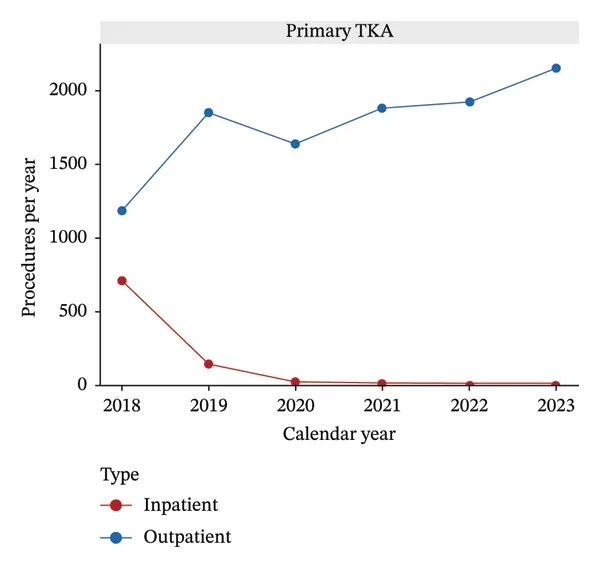
Inpatient versus outpatient volume postremoval of total knee arthroplasty from the Inpatient Only List.

### 3.6. Trends in Indications of rTKA

The composition of indications for rTKA for this orthopedic group aligned with national trends. Over the study period, on average, aseptic loosening (20.8%), PJI (36.2%), and breakage (14.8%) were the most common indications as illustrated in Table 2. The “Other Mechanical” category captured mechanical prosthesis‐ or implant‐related complications not otherwise categorized as loosening, instability, breakage, periprosthetic fracture, or bearing surface wear. The “Other” category represented nonmechanical or nonspecific prosthesis‐related complications that did not align with the predefined indication groups, encompassing fibrosis/scar‐related complications, prosthesis‐associated pain, and unspecified implant‐related complications.

**TABLE 2:** Distribution of indications for revision total knee arthroplasty from 2018 to 2023.

Revision indication	2018 (%)	2019 (%)	2020 (%)	2021 (%)	2022 (%)	2023 (%)	Average (%)
Loosening	21.08	14.43	17.88	30.35	24.68	16.30	20.80
Infection	37.25	37.11	39.11	29.35	41.14	34.24	36.16
Instability	14.71	7.73	3.35	1.00	2.53	2.72	5.54
Breakage	0.00	0.00	10.61	29.35	17.09	33.15	14.82
Periprosthetic fracture	3.92	8.25	8.38	3.48	7.59	8.15	6.52
Bearing surface wear	0.00	0.00	0.00	0.50	0.00	0.00	0.09
Other Mechanical	23.04	32.47	16.76	2.99	1.90	0.00	13.30
Other	0.00	0.00	3.91	2.99	5.06	5.43	2.77

## 4. Discussion

This study highlights important trends in the procedural volume of pTKAs and rTKAs in the context of a high volume private orthopedic practice before and after the onset of the COVID‐19 pandemic. Various predictions have been made in the literature regarding projected volume trends of both pTKAs and rTKAs in the United States extrapolated between the years 2008 and 2014. This paper focused on two time periods that highlight the impact of COVID‐19 on the average rate of procedural change. The first period, from 2018 to 2023, encompasses the COVID‐19 pandemic, while the second period, from 2020 to 2023, examines data from the peri‐COVID‐19 era. From 2018 to 2023, pTKA volume increased by 13.3%, corresponding to a compound annual growth rate of 2.53%. While including the significant reduction in procedural volume during the height of the pandemic, this average is similar to the average yearly growth predicted by Sloan et al. of 2.34% (1.56–3.78 CI) [6]. From 2020 to 2023, pTKA volume increased by 29.35%, corresponding to a compound annual growth rate of 8.96%. However, this temporal pattern does not establish that the COVID‐19 pandemic caused the subsequent increase. The observed growth may instead reflect a combination of deferred surgical demand, changes in referral volume, surgeon practice expansion, increased ambulatory capacity, and contemporaneous Medicare policy changes. Because these factors were not evaluated separately, their relative contributions to the observed volume trajectory cannot be determined.

Furthermore, reports from other centers suggest arthroplasty volume often did not return to prepandemic baselines even within two years of the initial COVID‐19 disruption, reflecting ongoing constraints in operating room capacity, staffing, and perioperative resources [14, 15]. In that context, the pronounced postpeak increase observed in this high‐volume private practice could potentially represent a distinct recovery pattern, potentially driven by regional differences in restrictions, referral dynamics, and the ability to rapidly restore elective capacity [16]. This trend is also consistent with a “catchup” effect from deferred care, in which accumulated demand for pTKA was compressed into a shorter timeframe once access returned [17]. If replicated in other settings, these findings could inform planning for rapid procedural‐volume rebounds after system‐wide disruptions, including proactive resource allocation and throughput optimization. They also potentially underscore the importance of continued surveillance of rTKA volume and outcomes, as sustained growth in primary arthroplasty volume may translate into a greater downstream revision burden.

Similar COVID‐19–related disruptions in arthroplasty volume have been reported internationally although the magnitude of decline and pace of recovery vary substantially across healthcare systems. A study from Romania demonstrated an abrupt reduction of up to 69% in elective hip and knee arthroplasty volume during the pandemic, accompanied by prolonged hospitalizations, increased rates of home discharge, and significant reductions in hospital revenue, with procedural volumes remaining below prepandemic levels through much of the recovery period [18]. Similar results were also reported globally, with data from Austria, Poland, and China demonstrating similar statistics [19–21]. These findings contrast with the accelerated postpandemic growth observed in the present study and suggest that system‐level factors may play a critical role in shaping recovery trajectories. Together, international data highlight that while COVID‐19 universally disrupted elective arthroplasty, postpandemic volume patterns are highly context‐dependent and may differ substantially between centralized national health systems and private practice–driven models of care.

In contrast to pTKAs, this study’s data did not mimic the increased rTKA volume trend predictions outlined in the literature and instead showed fluctuating increases and decreases in rTKA volume resulting in a net 1.9% increase in overall volume from 2018 to 2023. Schwartz et al. published a projection model based on inpatient procedural volume data generated between 2002 and 2014 and predicted a rTKA growth rate of 8.08% (4.88%–11.3%; linear vs. Poisson model) per year through the year 2030 [5]. The differences between the present study and previous studies may also be impacted by differing patient populations, the inclusion of data from the COVID‐19 era along with this practices’ specific patient population, changes in surgical techniques and technologies, implant design, and perioperative care [22].

In addition, the present study highlights how COVID‐19 was a potential contributor to the decrease in pTKA volume at the time of the pandemic onset, which corresponds with previous literature [12]. Reasons for this decrease are likely to include the implementation of lockdowns and the reallocation of medical resources [19, 23]. Revision TKAs saw a relatively small decrease in volume through the pandemic, possibly due to the often more severe and time sensitive etiology warranting the need for the procedure. This study’s data show a large increase in pTKA volume after the onset of the pandemic at a rate greater than the prepandemic volume. The initial increase can potentially be attributed to a backlog of cases unable to be completed during the lockdown time, but the continued increased rate of 46.68 cases per year may be due to other factors.

Several contemporaneous policy and practice changes may have contributed to the observed increase independently of the COVID‐19 pandemic. The removal of pTKA from the Medicare IPOL in 2018 expanded opportunities for outpatient surgery [24–26]. In addition, the practice began utilizing a local ambulatory surgery center in 2020, where pTKA volume increased from 32 procedures in 2020 to 229 procedures in 2023, while hospital‐based volume remained relatively stable (Figure 3). The coincident expansion of ambulatory capacity may, therefore, account for a portion of the overall procedural growth. However, the available data cannot determine whether the increased surgery‐center volume represented a shift from hospital‐based care, additional procedural capacity, or a true increase in underlying patient demand.

Comparison with the counterfactual prepandemic trajectory further qualifies the observed recovery. Actual pTKA volume remained below projected levels in 2021 and 2022 but exceeded the projected 2023 volume by 6.7% (2164 versus 2028 procedures). Across the entire postinterruption period, however, 7231 pTKAs were performed compared with 7497 projected. This pattern suggests an eventual rebound above the prepandemic trajectory rather than consistently elevated volume throughout the postpandemic period. These projections should be interpreted cautiously because they were derived from only nine prepandemic quarters and cannot account for contemporaneous changes in ambulatory capacity, referrals, or practice operations.

Unmeasured practice‐level changes may also have influenced procedural volume. Growth in the practice’s referral base, geographic catchment area, surgeon‐specific caseloads, clinic capacity, or operating room availability could have increased procedural volume regardless of the pandemic. Although the practice rapidly resumed elective surgery between Q2 and Q3 of 2020, this temporal association should not be interpreted as evidence that the pandemic caused the subsequent sustained growth. Regardless, the downstream effects–related early rebound were potentially beneficial to the practice including the maintenance of revenue, expanding to patient populations that likely would not have previously been accessible, and intangible factors such as improving patient trust and relationships by working through the difficult circumstances created by the pandemic.

Long‐term implications suggested by these findings include a potentially sustained increase in demand for pTKA after the COVID‐19 era, potentially reflecting both delayed care during periods of restricted elective surgery and an expanded pool of patients progressing to end‐stage osteoarthritis. If this rebound pattern is observed more broadly, it may be particularly relevant to private practices, where scheduling flexibility, ambulatory infrastructure, and referral dynamics can enable faster recovery and growth in elective volume [27, 28]. Practically, these trends suggest orthopedic groups should prepare for higher baseline demand and align capacity accordingly through operating room block‐time planning, anesthesia and nursing staffing models, clinic access and preoperative optimization pathways, implant and instrumentation supply‐chain coordination, and postoperative rehabilitation capacity. From a workforce standpoint, sustained volume growth also raises considerations around surgeon workload, advanced practice provider integration, and retention strategies to mitigate burnout during prolonged surges [29].

At the systems level, these data suggests that a re‐evaluation of widely cited TKA utilization projections derived entirely from prepandemic utilization trajectories could be warranted. Future forecasting models can start to incorporate the structural disruption introduced by COVID‐19 (including temporary cessation of elective procedures and subsequent backlog effects) as well as policy changes that can shift site‐of‐service and throughput such as Medicare’s IPOL. Updating projection methodologies to reflect these post‐2020 realities will be important not only for practice‐level planning but also for anticipating downstream needs in revision care, perioperative services, and regional access to arthroplasty as demand continues to evolve.

### 4.1. Limitations

This study has several limitations. Its single‐practice design and six‐year study period limit external validity and may not reflect broader regional or national arthroplasty trends. Although prepandemic growth and revision indications were broadly consistent with published national patterns, procedural volumes may have been influenced by practice‐specific patient populations, referral growth, surgeon practice expansion, changes in active staffing or operating room capacity, and increasing ambulatory surgery‐center use. These factors were not available for analysis, preventing separation of pandemic‐associated changes from underlying practice growth, expanded capacity, or shifts in site of service. Because procedures and revision indications were identified using ICD‐10 codes without chart‐level validation, coding errors, nonspecific codes, and multiple codes assigned to a single encounter may have resulted in misclassification. Furthermore, the aggregate dataset lacked patient‐level demographics, comorbidities, insurance status, and surgical indications that may have influenced procedural volume. The counterfactual projections were also based on only nine prepandemic quarters, limiting their precision and precluding more complex forecasting models. Accordingly, the findings should be interpreted as temporal associations rather than causal effects. Longer‐term, multicenter studies incorporating patient‐level and site‐of‐service data are needed to determine whether similar trends occurred more broadly.

## 5. Conclusions

Within this high‐volume private orthopedic practice, pTKA volume increased during the post‐COVID‐19 period following the initial pandemic‐related disruption. These findings suggest that the pandemic, together with contemporaneous policy and practice changes such as removal of TKA from the Medicare IPOL, may have influenced the observed volume trajectory. Longer‐term, multicenter studies are needed to determine whether similar patterns occurred more broadly and to inform updated volume projections and resource planning.

## Funding

No funding was received for this manuscript.

## Conflicts of Interest

The authors declare no conflicts of interest.

## Supporting Information

Additional supporting information can be found online in the Supporting Information section.

## Supporting information


**Supporting Information** The STROBE checklist used to guide and assess the reporting of this observational study is provided as Supporting File 1. List of procedural diagnosis codes for each revision indication for revision total knee arthroplasty included in the study.

## Data Availability

Research data are not shared.
