# Supplementary material for: Reevaluating Projections: Total Knee Arthroplasty Volume Before and After COVID‐19 in a Single Orthopedic Practice
Source: Adv Orthop. 2026 Oct 6;2026:5527598. doi: 10.1155/aort/5527598 (PMC13639843; doi:10.1155/aort/5527598)
Supplement: Supplementary file 1 [file AORT-2026-5527598-s001.zip › STROBE Statement (1).docx]

**STROBE Statement—checklist of items that should be included in reports of observational studies**

*Study: Revaluating Projections: How COVID-19 Altered the Landscape of Total Knee Arthroplasty Volume in a Single Orthopedic Practice*

| **Section/topic** | **Item No** | **Recommendation** | **Reported on page/section** |
| --- | --- | --- | --- |
| **Title and abstract** | **1** | **(*a*) Indicate the study’s design with a commonly used term in the title or the abstract** | **Abstract, p. 2 (retrospective design identified)** |
|  |  | **(*b*) Provide in the abstract an informative and balanced summary of what was done and what was found** | **Abstract, pp. 2–3** |
| **Introduction** |  |  |  |
| **Background/rationale** | **2** | **Explain the scientific background and rationale for the investigation being reported** | **Introduction, pp. 3–4** |
| **Objectives** | **3** | **State specific objectives, including any prespecified hypotheses** | **Introduction, p. 5** |
| **Methods** |  |  |  |
| **Study design** | **4** | **Present key elements of study design early in the paper** | **Methods 2.1, p. 5** |
| **Setting** | **5** | **Describe the setting, locations, and relevant dates, including periods of recruitment, exposure, follow-up, and data collection** | **Methods 2.1 and 2.3, pp. 5 and 7** |
| **Participants** | **6** | **(*a*) *Cohort study*—Give the eligibility criteria, and the sources and methods of selection of participants. Describe methods of follow-up**  ***Case-control study*—Give the eligibility criteria, and the sources and methods of case ascertainment and control selection. Give the rationale for the choice of cases and controls**  ***Cross-sectional study*—Give the eligibility criteria, and the sources and methods of selection of participants** | **Methods 2.1–2.2, pp. 5–6** |
|  |  | **(*b*) *Cohort study*—For matched studies, give matching criteria and number of exposed and unexposed**  ***Case-control study*—For matched studies, give matching criteria and the number of controls per case** | **Not applicable—unmatched procedure cohort** |
| **Variables** | **7** | **Clearly define all outcomes, exposures, predictors, potential confounders, and effect modifiers. Give diagnostic criteria, if applicable** | **Methods 2.2–2.3, pp. 6–7** |
| **Data sources/ measurement** | **8*** | **For each variable of interest, give sources of data and details of methods of assessment (measurement). Describe comparability of assessment methods if there is more than one group** | **Methods 2.2, p. 6; Supplemental Materials, p. 22** |
| **Bias** | **9** | **Describe any efforts to address potential sources of bias** | **Methods 2.2–2.4, pp. 6–8; Limitations 4.1, pp. 18–19** |
| **Study size** | **10** | **Explain how the study size was arrived at** | **Methods 2.3, p. 7 (all eligible procedures included)** |
| **Quantitative variables** | **11** | **Explain how quantitative variables were handled in the analyses. If applicable, describe which groupings were chosen and why** | **Methods 2.3–2.4, p. 7** |
| **Statistical methods** | **12** | **(*a*) Describe all statistical methods, including those used to control for confounding** | **Methods 2.4, pp. 7–8** |
|  |  | **(*b*) Describe any methods used to examine subgroups and interactions** | **Not applicable—no subgroup or interaction analyses** |
|  |  | **(*c*) Explain how missing data were addressed** | **Methods 2.2, p. 6 (no imputation; indication-specific exclusions described)** |
|  |  | **(*d*) *Cohort study*—If applicable, explain how loss to follow-up was addressed**  ***Case-control study*—If applicable, explain how matching of cases and controls was addressed**  ***Cross-sectional study*—If applicable, describe analytical methods taking account of sampling strategy** | **Not applicable—aggregate procedural series without patient follow-up** |
|  |  | **(*e*) Describe any sensitivity analyses** | **Methods 2.4 and Results 3.3 (revised sensitivity analyses; update final pages)** |

| **Section/topic** | **Item No** | **Recommendation** | **Reported on page/section** |
| --- | --- | --- | --- |
| **Results** |  |  |  |
| **Participants** | **13*** | **(a) Report numbers of individuals at each stage of study—eg numbers potentially eligible, examined for eligibility, confirmed eligible, included in the study, completing follow-up, and analysed** | **Methods 2.1, p. 5; Results 3.1, p. 8; Table 1, p. 9** |
|  |  | **(b) Give reasons for non-participation at each stage** | **Not applicable—all eligible procedures included; missing indications addressed in Methods 2.2, p. 6** |
|  |  | **(c) Consider use of a flow diagram** | **Not applicable—no multistage enrollment; flow diagram not required** |
| **Descriptive data** | **14*** | **(a) Give characteristics of study participants (eg demographic, clinical, social) and information on exposures and potential confounders** | **Not available in the aggregate dataset; acknowledged in Limitations 4.1** |
|  |  | **(b) Indicate number of participants with missing data for each variable of interest** | **Methods 2.2, p. 6** |
|  |  | **(c) *Cohort study*—Summarise follow-up time (eg, average and total amount)** | **Not applicable—no individual longitudinal follow-up** |
| **Outcome data** | **15*** | ***Cohort study*—Report numbers of outcome events or summary measures over time** | **Results 3.1–3.6, pp. 8–13; Tables 1–2 and Figures 1–4** |
|  |  | ***Case-control study—*Report numbers in each exposure category, or summary measures of exposure** | **Not applicable—cohort study** |
|  |  | ***Cross-sectional study—*Report numbers of outcome events or summary measures** | **Not applicable—cohort study** |
| **Main results** | **16** | **(*a*) Give unadjusted estimates and, if applicable, confounder-adjusted estimates and their precision (eg, 95% confidence interval). Make clear which confounders were adjusted for and why they were included** | **Results 3.3 and Figure 2 (revised segmented-regression estimates; update final pages)** |
|  |  | **(*b*) Report category boundaries when continuous variables were categorized** | **Methods 2.3, p. 7 (quarter and pandemic-period boundaries)** |
|  |  | **(*c*) If relevant, consider translating estimates of relative risk into absolute risk for a meaningful time period** | **Not applicable—no relative-risk estimates** |
| **Other analyses** | **17** | **Report other analyses done—eg analyses of subgroups and interactions, and sensitivity analyses** | **Results 3.3 (revised sensitivity analyses; update final pages)** |
| **Discussion** |  |  |  |
| **Key results** | **18** | **Summarise key results with reference to study objectives** | **Discussion, pp. 14–18; Conclusions, p. 19** |
| **Limitations** | **19** | **Discuss limitations of the study, taking into account sources of potential bias or imprecision. Discuss both direction and magnitude of any potential bias** | **Limitations 4.1, pp. 18–19** |
| **Interpretation** | **20** | **Give a cautious overall interpretation of results considering objectives, limitations, multiplicity of analyses, results from similar studies, and other relevant evidence** | **Discussion, pp. 14–18; Conclusions, p. 19** |
| **Generalisability** | **21** | **Discuss the generalisability (external validity) of the study results** | **Discussion and Limitations, pp. 14–19** |
| **Other information** |  |  |  |

*Give information separately for cases and controls in case-control studies and, if applicable, for exposed and unexposed groups in cohort and cross-sectional studies.

**Note:** An Explanation and Elaboration article discusses each checklist item and gives methodological background and published examples of transparent reporting. The STROBE checklist is best used in conjunction with this article (freely available on the Web sites of PLoS Medicine at http://www.plosmedicine.org/, Annals of Internal Medicine at http://www.annals.org/, and Epidemiology at http://www.epidem.com/). Information on the STROBE Initiative is available at www.strobe-statement.org.
