# Supplementary material for: Reevaluating Projections: Total Knee Arthroplasty Volume Before and After COVID‐19 in a Single Orthopedic Practice
Source: Adv Orthop. 2026 Oct 6;2026:5527598. doi: 10.1155/aort/5527598 (PMC13639843; doi:10.1155/aort/5527598)
Supplement: Supplementary file 1 [file AORT-2026-5527598-s001.zip › Supplemental materials updated .docx]

**Supplementary material:**

**Revision Total Knee Arthroplasty Diagnosis Codes**

**Loosening**

**Mechanical loosening of internal left knee prosthetic joint, initial encounter [T84.033A]**

**Mechanical loosening of internal left knee prosthetic joint, subsequent encounter [T84.033D]**

**Mechanical loosening of internal right knee prosthetic joint, initial encounter [T84.030A]**

**Mechanical loosening of internal right knee prosthetic joint, initial encounter [T84.032A]**

**Infection**

**Infection and inflammatory reaction due to internal left knee prosthesis, initial encounter [T84.54XA]**

**Infection and inflammatory reaction due to internal left knee prosthesis, subsequent encounter [T84.54XD]**

**Infection and inflammatory reaction due to internal right knee prosthesis, initial encounter [T84.53XA]**

**Infection and inflammatory reaction due to internal right knee prosthesis, subsequent encounter [T84.53XD]**

**Infection and inflammatory reaction due to other internal joint prosthesis, initial encounter [T84.59XA]**

**Infection and inflammatory reaction due to other internal joint prosthesis, subsequent encounter [T84.59XD]**

**Infection and inflammatory reaction due to other internal orthopedic prosthetic devices, implants and grafts, subsequent encounter [T84.7XXD]**

**Instability**

**Instability of internal left knee prosthesis, initial encounter [T84.023A]**

**Instability of internal right knee prosthesis, initial encounter [T84.022A]**

**Breakage**

**Broken internal left knee prosthesis, initial encounter [T84.013A]**

**Broken internal left knee prosthesis, subsequent encounter [T84.013D]**

**Broken internal right knee prosthesis, initial encounter [T84.012A]**

**Broken internal right knee prosthesis, subsequent encounter [T84.012D]**

**Periprosthetic fracture**

**Periprosthetic fracture around internal prosthetic left knee joint, initial encounter [M97.12XA]**

**Periprosthetic fracture around internal prosthetic right knee joint, initial encounter [M97.11XA]**

**Periprosthetic fracture around internal prosthetic right knee joint, sequela [M97.11XS]**

**Periprosthetic fracture around internal prosthetic right knee joint, subsequent encounter [M97.11XD]**

**Periprosthetic osteolysis of internal prosthetic right knee joint, initial encounter [T84.052A]**

**Bearing surface wear**

**Wear of articular bearing surface of internal prosthetic right knee joint, initial encounter [T84.062A]**

**Other Mechanical**

**Other mechanical complication of internal left knee prosthesis, initial encounter [T84.091A]**

**Other mechanical complication of internal left knee prosthesis, initial encounter [T84.093A]**

**Other mechanical complication of internal right knee prosthesis, initial encounter [T84.090A]**

**Other mechanical complication of internal right knee prosthesis, initial encounter [T84.092A]**

**Other mechanical complication of other internal orthopedic devices, implants and grafts, initial encounter [T84.498A]**

**Other mechanical complication of other internal orthopedic devices, implants and grafts, sequela [T84.498S]**

**Other**

**Acquired absence of left knee [Z89.522]**

**Acquired absence of right knee [Z89.521]**

**Displaced fracture of right tibial tuberosity, initial encounter for closed fracture [S82.151A]**

**Displaced longitudinal fracture of right patella, initial encounter for closed fracture [S82.021A]**

**Displaced transverse fracture of right patella, initial encounter for closed fracture [S82.031A]**

**Pain due to internal orthopedic prosthetic devices, implants and grafts, initial encounter [T84.84XA]**

**Presence of right artificial knee joint [Z96.651]**

**Unilateral post-traumatic osteoarthritis, left knee [M17.32]**

**Unilateral primary osteoarthritis, left knee [M16.12]**

**Unilateral primary osteoarthritis, left knee [M17.12]**

**Unilateral primary osteoarthritis, right knee [M17.11]**
